# 34380 Cadherin complexes recruit PIWIL2 to suppress transposons and pro-tumorigenic transformation

**DOI:** 10.1017/cts.2021.432

**Published:** 2021-03-30

**Authors:** Alyssa Risner, Joyce Nair-Menon, Colin McDowell, Vamsi Gangaraju, Antonis Kourtidis

**Affiliations:** ^1^ Department of Regenerative Medicine and Cell Biology; ^2^ Department of Cell and Molecular Pharmacology and Experimental Therapeutics; ^3^ Department of Biochemistry and Molecular Biology, Medical University of South Carolina, 173 Ashley Avenue, Charleston, SC 29425

## Abstract

ABSTRACT IMPACT: This study has uncovered a novel surprising mechanism involving the epithelial adherens junctions and transposon regulation that can deepen our understanding of tumorigenesis. OBJECTIVES/GOALS: Recent studies show that genomic instability in 50% of tumors can be attributed to increased transposon activity, but the the reasons for this activity are unknown. We have evidence of a novel mechanism linking adherens junctions with transposon regulation. We hypothesize that adherens junctions suppress transposons to maintain genomic integrity. METHODS/STUDY POPULATION: We observed co-localization of PIWIL2 with adherens junction components of well differentiated epithelial breast, kidney and colon cell lines MCF10A, MDCK and CACO2, respectively, through immunofluorescence staining, confocal microscopy, and co-immunoprecipitation studies. Breast cancer cell lines MCF7 and MDA-231 were also observed using immunofluorescence to determine the localization of PIWIL2 in cancer cell lines. shRNA knockdown of PIWIL2 in MCF10A cells, followed by western blot, immunofluorescence, and qRT-PCR was performed to confirm the knockdown, observe if transposons were upregulated, and determine the extent of DNA damage to the genome by the marker gamma-H2AX. RNA-seq will be performed to determine piRNA sequences and possible targets of PIWIL2. RESULTS/ANTICIPATED RESULTS: Our data have revealed an interaction of E-cadherin and p120 catenin, core components of adherens junctions, with PIWIL2, a member of the Argonaute family of proteins and a key component of the piRNA processing pathway that is responsible for transposon silencing. piRNAs (PIWI-interacting RNAs) are a distinct class of small RNAs that bind to PIWI proteins, and aid in transposon degradation. We found co-localization of PIWIL2 with E-cadherin and p120 catenin at adherens junctions of well-differentiated epithelial cells, whereas this association was lost in cancer cells. Furthermore, our data show that E-cadherin depletion results in mis-localization of PIWIL2 and TDRD1, another member of the PIWI complex. E-cadherin depletion also results in upregulation of transposons and ?-H2AX, an indicator of DNA damage. DISCUSSION/SIGNIFICANCE OF FINDINGS: Since both loss of junctional integrity and increased transposon activity are universal events in cancer, this study has the potential to further our understanding of the causes of tumorigenesis. Understanding the mechanisms of transposon regulation has the potential to lead to a therapeutic target in the future.

